# Causes of mortality in humanitarian crises requiring coordinated response: a scoping review protocol

**DOI:** 10.1136/bmjopen-2025-110528

**Published:** 2026-10-07

**Authors:** Paula Christen, Nisreen Shehada, Nada Abdelmagid, Ruwan Ratnayake, Hanan Abukmail, Arran Hamlet, Catherine McGowan, Oliver J Watson, Bhargavi Rao

**Affiliations:** 1 WHO Collaborating Centre for Infectious Disease Modelling, MRC Centre for Global Infectious Disease Analysis, School of Public Health, Imperial College London, London, UK; 2 Abdul Latif Jameel Institute for Disease and Emergency Analytics, School of Public Health, Imperial College London, London, UK; 3 Center for Epidemiological Modelling and Analysis (CEMA), University of Nairobi, Nairobi, Kenya; 4 Department of Infectious Disease Epidemiology and International Health, Faculty of Epidemiology and Population Health, London School of Hygiene & Tropical Medicine, London, UK; 5 MRC Centre for Global Infectious Disease Analysis, School of Public Health, Imperial College London, London, UK; 6 Epidemic Preparedness and Response, Global Health Programs, PATH, London, UK; 7 Global Health Department, Faculty of Public Health Policy, London School of Hygiene & Tropical Medicine, London, UK; 8 Manson Unit, Médecins Sans Frontières (MSF), London, UK

**Keywords:** EPIDEMIOLOGY, Mortality, Review

## Abstract

**Abstract:**

**Introduction:**

Mortality patterns in humanitarian crises are shaped by intersecting factors including violence, displacement, health system disruption and environmental shocks. While crude and under-five mortality rates are often tracked, less is known about specific causes of death across diverse crisis contexts. Existing guidance informing humanitarian response is largely informed by outdated evidence and does not account for demographic, epidemiological and geopolitical shifts over the past two decades. This review aims to synthesise available data on cause-specific mortality in humanitarian settings to inform improved monitoring, policy and response.

**Methods and analysis:**

We will conduct a scoping review of peer-reviewed and grey literature reporting on causes of death in humanitarian crises between 2015 and 2025. Eligible studies will include crisis-affected populations in locations with an active Humanitarian Response Plan during analysis. We will search Medline, Embase and Global Health, and screen organisational datasets from key humanitarian agencies. A large language model will be used to assist with title and abstract screening. Full-text screening and data extraction will be conducted manually by two reviewers. Methodological quality will be assessed using study-type-specific appraisal tools. Data will be extracted using a standardised plan and synthesised narratively.

**Ethics and dissemination:**

Ethics approval is not required as this study uses publicly available data. Findings will be submitted to a peer-reviewed journal and disseminated via policy briefs, academic presentations and targeted stakeholder engagement.

**STRENGTHS AND LIMITATIONS OF THIS STUDY**

- This review will synthesise evidence on cause-specific mortality in humanitarian crises globally, incorporating both quantitative and qualitative studies, allowing for a broad synthesis of diverse mortality data collection approaches, including surveys, surveillance and verbal autopsy.
- Large language model–assisted title and abstract screening increases sensitivity, with human verification of all decisions.
- Findings may be limited by inconsistent reporting standards, geographic gaps and heterogeneity of study designs.

## Introduction

 Over the past 30 years, humanitarian crises have become increasingly complex, prolonged and severe. They are driven by armed conflict, natural hazards and climate-related events, often in combination, and are frequently characterised by weakened protection systems and violations of international humanitarian law. Although the events that trigger such crises are highly heterogeneous, they tend to converge on a common set of crisis-emergent risk factors through which health and mortality are affected.^1^ These include disruption of routine health services and reduced access to quality care, deterioration of water, sanitation and hygiene (WASH) conditions, food insecurity and malnutrition, displacement, and damage to essential infrastructure.

These factors give rise to both direct and indirect mortality, and their relative contribution varying across crises and over time. Alongside the direct loss of life from injuries and violence, disruption of health services, as seen in the recent and ongoing crises in Haiti, Pakistan, Gaza and Tigray, erodes primary care, interrupts vaccination and treatment for chronic conditions, and increases the risk of infectious disease outbreaks.^2^ Together with deteriorating WASH conditions, food insecurity, displacement and infrastructure breakdown, these disruptions can contribute substantially to excess morbidity and preventable mortality, including long after the initial shock.

### Gaps in the literature

Despite the increasing complexity of humanitarian crises, the evidence base informing decision-making remains limited and fragmented, and may not comprehensively reflect varying causes of death patterns across affected populations and contexts. Foundational resources, such as the Sphere Handbook, continue to shape response priorities and mortality surveillance practices. However, much of this guidance is based on evidence that is more than two decades old^3 4^ and does not reflect recent changes in crisis contexts, including urban warfare, demographic transitions, the growing burden of non-communicable diseases and climate-related risks.^5^ As a result, current frameworks may not adequately capture contemporary patterns of mortality across affected populations.

Our preliminary searches of PROSPERO and Medline(R) did not identify any systematic or scoping review in the past two decades that has examined causes of mortality across all age groups in humanitarian crises globally. This gap persists despite the importance of such data for priority setting, needs assessment and resource allocation.^1 6^ In particular, there is no comprehensive synthesis of cause-specific mortality in crises driven by climate-related events or natural disasters, such as droughts, floods or earthquakes, which are increasing in frequency and severity.^7^ Studies in conflict-affected and displaced populations also report a high burden of preventable conditions, including measles, diarrhoeal diseases, acute respiratory infections, malaria and malnutrition. For example, findings from the Democratic Republic of the Congo illustrate similar patterns across age groups, with febrile illness, diarrhoea and respiratory infections among leading causes of death.^8^ At the same time, mortality among older adults increasingly reflects both communicable and non-communicable diseases, including cardiovascular conditions,^9 10^ while women and children face elevated risks in conflict settings.^11 12^ Mortality data are primarily generated through surveys, surveillance systems and facility reporting, each with important limitations. Yet, these approaches often lack reliable attribution of cause of death and may exclude insecure areas or displaced populations.^13 14^ Although recent studies have estimated excess mortality in settings such as Tigray, Yemen, Syria, Gaza and Azerbaijan,^15–20^ few have systematically examined cause-specific mortality or assessed the adequacy of available data. There remains a clear need for a comprehensive synthesis of causes of death and their measurement in humanitarian crises.

### Aims

The objective of this scoping review is to identify and synthesise evidence on leading causes of mortality in humanitarian crises, with the goal of informing resource allocation and priority setting. Specifically, we aim to:

1. Identify the principal causes of mortality among crisis-affected populations.
2. Assess how mortality patterns differ by demographic group, by population group (internally displaced versus resident populations) and by crisis type (box 1).
3. Distinguish between direct and indirect causes of mortality and characterise how their relative contributions shift along the acute-to-protracted crisis spectrum and across underlying risk-factor pathways (health services, WASH, food security and nutrition).
4. Document how cause of death is measured and thereby develop a typology of measurement approaches.

## Methods and analysis

To ensure conceptual clarity and consistency, we use the definitions listed in Text box 1 throughout the protocol.

Box 1 **Key definitions used in this review**

- **Cause-specific mortality:** Deaths attributed to a specific cause or group of causes, as classified in the original source.
- **All-cause mortality:** Total number of deaths from all causes over a defined period, without restriction to specific causes.
- **Excess mortality**: The number of deaths above what would be expected based on pre-crisis or baseline mortality levels, accounting for trends and seasonality where possible.
- **Crisis type:** The nature of the humanitarian crisis under study, classified as (1) armed conflict; (2) natural hazard, including climate-related events such as drought, flood and storm; (3) epidemic; or (4) complex emergency, where two or more of these overlap. Crises are additionally classified as acute or protracted (see below).
- **Acute crisis:** A humanitarian crisis characterised by a sudden onset or rapid escalation over a relatively short period, often associated with immediate threats to life, health and access to essential services.
- **Protracted crisis:** A humanitarian crisis in which a significant proportion of the population remains exposed to sustained or recurrent risks over an extended period, often with prolonged displacement, disrupted services and long-term humanitarian need.

### Inclusion and exclusion criteria

#### Inclusion criteria

Studies will be included if they meet all of the following criteria:

- **Population and setting**
  - Studies must examine crisis-affected populations in humanitarian settings.
  - Eligible populations include internally displaced persons (IDPs), crisis-affected host populations, and other groups resident in the crisis-affected setting and directly affected by the crisis.
  - Studies must be conducted in countries with a Humanitarian Response Plan (HRP) issued since 2015 and prior to data collection. HRPs are country-level strategic plans coordinated by the UN Office for the Coordination of Humanitarian Affairs (OCHA), developed jointly by humanitarian partners in response to a crisis requiring international assistance. An active HRP indicates that a country is experiencing a humanitarian situation of sufficient scale and severity to warrant a coordinated international response and therefore serves as a practical, widely recognised marker of countries in crisis. A predefined list of eligible countries, based on HRP presence, is provided in online supplemental table 3.
- **Exposure**
  - Studies must involve populations exposed to a humanitarian crisis, as classified in box 1:
    - Armed conflict.
    - Natural hazards, including climate-related events.
    - Epidemics.
    - Complex emergencies.
- **Outcomes**
  - Studies must report on cause-specific mortality or provide disaggregated information on causes of death.
  - Eligible outcomes include, but are not limited to:
    - Cause-specific mortality estimates or rankings, crude and across all age groups.
    - All-cause mortality measures, where disaggregated by cause of death.
    - Age-specific mortality rates, including for children under 5 years (0–4 years), children and adolescents (5–19 years), adults (20–64 years) and older adults (≥65 years).
    - Age-standardised mortality rates, where reported.
    - Commonly reported mortality indicators, such as:
      - Crude Death Rate (CDR).
      - Under-five Death Rate (U5DR).
      - Under-five Mortality Rate (U5MR).
      - Infant Mortality Rate (IMR).
      - Maternal Mortality Ratio (MMR).
- **Study types and data sources**
  - No restrictions will be placed on the methods used to estimate mortality or assign cause of death. Both quantitative and qualitative assessments (eg, verbal autopsy, surveillance data or expert attribution) will be included, provided that deaths are attributed to specific causes within a humanitarian crisis context.
- Time period
  - Studies must report on data collected from 2015onwards.
- Language
  - Studies published in any language will be eligible.Screening and data extraction of non-Englishstudieswill be supported by multilingual members ofthe review team and collaborators where available.Where necessary, machine translation tools will beused to assist with eligibility assessment and data extraction. Inclusion of non-Englishstudies willbe subject to available translation capacity, and anylimitations in language coverage will be documentedand reported.

### Exclusion criteria

Studies will be excluded if they meet any of the following criteria:

- Focus on populations residing in non-crisis-affected areas within crisis-affected countries (eg, studies conducted in stable urban areas such as Lagos without disaggregation to crisis-affected regions).
- Do not demonstrate a clear link to a humanitarian crisis context.
- Do not report or disaggregate cause of death.
- Are based solely on pre-crisis data or focus exclusively on pre-crisis time periods.

### Information sources

Databases (Embase, Medline and Global Health) of peer-reviewed studies will be searched. These were selected for their comprehensive coverage of biomedical, public health and global health literature relevant to humanitarian settings. Grey literature will include situation reports, humanitarian needs assessments, internal mortality surveys and operational datasets developed by UN agencies (eg, World Health Organization (WHO), United Nations High Commissioner for Refugees (UNHCR)), non-governmental organizations (NGO) (eg, Médecins Sans Frontières (MSF)/Epicentre) and humanitarian coordination platforms. Further details are presented in the following section on the search strategy.

### Search strategy

We first confirmed the absence of existing or ongoing systematic reviews by searching the PROSPERO database (see online supplemental table 1) and the Open Science Framework (OSF) database. A preliminary search of peer-reviewed literature was conducted in Medline via the Ovid platform on 02 September 2025, yielding 38 137 records published between 2015 and September 2025. This initial Medline search strategy (see online supplemental table 2) will be refined in collaboration with a librarian and adapted for use in additional databases, including Embase and Global Health. The final strategy will be assessed using the Peer Review of Electronic Search Strategies (PRESS) checklist to ensure completeness and rigour.

To supplement the database searches, reference lists of included studies will be screened. Authors of eligible studies may be contacted to clarify data or request additional information where appropriate.

Grey literature will be identified through targeted follow-up with key humanitarian initiatives that have aimed to measure causes of death in crisis settings. Relevant agencies will be contacted to inquire about operational data collected through methods such as community-based mortality surveillance, surveys incorporating modules on cause of death and clinical data from health facilities (eg, MSF Operational Centre Brussels). Examples of relevant initiatives include:

- Verbal autopsy through mortality surveillance.^21^
- UNICEF Real-Time Monitoring of U5 mortality.^22^
- International Network for the Demographic Evaluation of Populations and their Health (INDEPTH) surveillance network.^23^
- WHO verbal autopsy.^24 25^
- Reproductive, Maternal, Newborn and Child Health surveillance in Cox’s Bazar,^26^ and Afghanistan by United Nations Population Fund (UNFPA) and Centers for Disease Control and Prevention (CDC).

Anticipated challenges in identifying relevant grey literature include language barriers, lack of public availability and access restrictions where data are held under confidentiality or require formal data-sharing agreements. To address these, we will: (1) seek support from multilingual collaborators to screen and translate non-English materials; (2) leverage professional networks and existing partnerships to initiate data-sharing discussions early; and (3) prioritise open-access and publicly archived materials while pursuing formal agreements for restricted datasets.

### Citation management

All records identified through the search strategy will be managed by a single reviewer. Search results will be exported and uploaded into a reference management platform (Covidence), where duplicate entries will be automatically identified and removed prior to screening.

### Selection process

Before screening, the review team will pilot the eligibility and exclusion criteria on a random sample of retrieved records. Prior to screening, the large language model (LLM)-assisted approach will be piloted on a random sample of abstracts (n=300 per batch), which will be independently reviewed by two human reviewers to establish a reference standard. Agreement between the LLM and the human reviewers will be assessed, with a ≥98% concordance threshold used as a benchmark to refine the screening approach. This process will be conducted iteratively, with successive batches used to refine the LLM prompts and classification rules until the predefined agreement threshold is achieved.

Previous studies have shown that single-human abstract screening may miss a small proportion of eligible studies (approximately 13%).^27^ Emerging evidence suggests that LLM-assisted screening approaches can achieve high sensitivity in controlled pilot settings, although findings remain context-specific and require validation.^28^ Following piloting, the LLM will conduct the initial title and abstract screening to identify eligible studies. This approach can substantially reduce screening workload but carries risks, including the potential misinterpretation of nuanced eligibility criteria. We will mitigate risks associated with automated screening by first validating LLM performance against human decisions on a subset of abstracts. Only after achieving high agreement (≥98%) is the model applied to the full dataset. Further, records classified as ‘include’, as well as those deemed uncertain or borderline by the LLM, will be reviewed by human reviewers. Conservative inclusion thresholds will be applied to favour inclusion in cases of uncertainty. In addition, two LLMs (DeepSeek and OpenAI Codex) will be applied in parallel for title and abstract screening. Discrepancies between the models, specifically records included by one model but excluded by the other, will be manually reviewed by human reviewers to maximise screening sensitivity and minimise the risk of missing eligible studies.

Reviewers will then independently perform full-text screening of all records identified as potentially eligible. The LLM will not be used for full-text screening. Discrepancies at either the LLM screening or full-text review stage will be resolved by the same reviewers and/or through broader team consensus.

To address potential duplication, eligible records will be examined for their publication date and underlying data source. If multiple studies are derived from the same dataset, the review will include either the most recent publication or the study with the longest follow-up period. Reasons for exclusion will be documented and presented for all records excluded at the full-text review stage.

### Data collection process

#### Critical appraisal of individual studies

Two reviewers will independently undertake critical appraisal of the included studies. We will apply study-type-specific quality appraisal tools^29^ to assess the methodological rigour of included studies:

- For quantitative studies, we will use the Appraisal Tool for Cross-Sectional Studies (AXIS), the Critical Appraisal Skills Programme (CASP) cohort study checklist and the Institute of Health Economics (IHE) Quality Appraisal Tool for longitudinal studies.
- For mixed-methods studies, we will use the Mixed Methods Appraisal Tool *(*MMAT).

Any potential disagreements will be solved by consensus within the review team.

### Data extraction

Full-text data extraction, following the data extraction plan (online supplemental table 4), will initially be conducted by two reviewers on a subset of manuscripts to ensure consistency. The data extraction approach will remain adaptive, acknowledging the heterogeneity in how causes of death are reported across sources. In some cases, mortality rate estimates will be derived not from standardised, quantitative datasets, but from qualitative descriptions or summary assessments in the original reports. In such instances, clinical or domain experts will interpret the available narrative information to approximate quantitative values or to classify the severity of outcomes. Any discrepancies will be resolved through discussion within the review team. Extracted data will be managed using Qualtrics.

### Data synthesis and analysis

#### Synthesis

We will categorise studies by crisis type and by acute or protracted status (box 1) to summarise the characteristics of included studies, critical appraisal scores, the relative contribution of each cause of death and other key findings. Given the heterogeneity of crisis types, findings will be stratified by crisis context to ensure meaningful interpretation. Results will be presented narratively and, where appropriate, using tables and figures.

Where appropriate, a mixed-methods data extraction approach will be employed, combining structured fields with qualitative coding, particularly for findings reported narratively.

When studies do not report a comparator group for mortality, we will draw on external reference estimates to contextualise findings and estimate excess or attributable mortality. Pre-crisis or baseline mortality will, where possible, be obtained from the same geographic area or population using historical surveillance data, vital registration systems or previous surveys. Where such local data are unavailable, we will use non-affected populations with similar demographic, socioeconomic and health system characteristics, identified through nationally representative surveys or administrative data. Key reference sources will include the Demographic and Health Surveys (DHS), Multiple Indicator Cluster Surveys (MICS), World Bank population and health statistics, and the Global Burden of Disease (GBD) study. Where appropriate, we will also draw on harmonised and collated datasets such as the WHO Mortality Database, the Health Equity Assessment Toolkit (HEAT) and UNICEF child health and mortality datasets, as well as other comparable national and international data sources.

In some cases, cause-specific mortality estimates from other humanitarian settings may be used as contextual comparators. Comparator selection will prioritise similarity in population characteristics, time period and measurement methods to minimise bias.

Given the likely heterogeneity in study designs, data sources and reporting formats, statistical pooling of results may not be feasible. Instead, we will undertake a narrative synthesis to triangulate data, guided by the Cochrane Handbook for Systematic Reviews of Interventions, drawing out common patterns, discrepancies and thematic insights across studies.

### Presenting and reporting results

The review will adhere to Preferred Reporting Items for Systematic reviews and Meta-Analyses extension for Scoping Reviews (PRISMA-ScR).^30^ A PRISMA-ScR flow diagram will detail the process of study identification, screening and inclusion. The discussion will highlight the main findings, examine limitations of both the included studies and the review itself, and consider their relevance for policy, data collection tools, mortality survey designs, programming and future research.

### Patient and public involvement

Patients and the public were not involved in the design, conduct, reporting or dissemination plans of this scoping review protocol.

## Discussion

This review will offer a structured synthesis of the best available evidence on causes of mortality across humanitarian settings, capturing both peer-reviewed and operational data. Given the heterogeneity of study designs, populations and mortality surveillance systems in crises, a flexible and inclusive review methodology is essential. By incorporating both quantitative and qualitative sources, the study is well positioned to identify under-reported patterns of mortality and assess the strength of attribution methods currently in use.

The inclusion of studies with different methodologies and data collection approaches will allow for a richer understanding of mortality drivers, but it also introduces challenges related to comparability. Studies may differ in how deaths are classified (eg, International Classification of Diseases (ICD) coding vs expert judgement), the timeframes over which data are collected and whether mortality is reported at individual, household or community levels. This variation may limit the generalisability of findings settings, though the review will aim to account for this through structured extraction and narrative synthesis.

From a policy perspective, the findings of this review may support improvements in mortality surveillance design, especially in protracted emergencies where rapid and reliable mortality data are essential but hard to obtain. Understanding how causes of death are currently captured, along with where they are overlooked, can help refine guidance and standards for future crisis response monitoring. Findings may also inform updates to existing public health triggers used for initiating scaled-up interventions or anticipatory action. Similarly, findings may also inform humanitarian funding mechanisms, resource mobilisation or inter-agency coordination structures, in particular in contexts where surveillance data availability is limited.

Finally, while the review seeks to be global in scope, it may be constrained by geographic imbalances in data availability. For example, crises in regions with better-resourced surveillance or greater donor visibility may be disproportionately represented in the evidence base. The review will document such limitations systematically and highlight areas where primary data collection efforts are most urgently needed.

## Ethics and dissemination

Ethics approval is not required as this study uses publicly available data. The findings will be submitted for publication in a peer-reviewed scientific journal and presented at relevant conferences in the field, as well as in meetings with key agencies engaged in collecting mortality data in complex emergencies and practitioners working in low-resource humanitarian contexts. We will also engage with norm-setting entities such as Sphere, WHO and MSF to ensure the results inform relevant technical guidance, standards and operational tools. Plain-language summaries of the key findings will be prepared and disseminated through existing peer and social networks. Further, we will engage with local humanitarian organisations and/or affected community representatives during dissemination activities to maximise local impact and operational relevance.

## Supplementary material

10.1136/bmjopen-2025-110528 online supplemental file 1
