# Supplementary material for: Causes of mortality in humanitarian crises requiring coordinated response: a scoping review protocol
Source: BMJ Open. 2026 Oct 7;16(10):e110528. doi: 10.1136/bmjopen-2025-110528 (PMC13646983; doi:10.1136/bmjopen-2025-110528)
Supplement: Supplementary file 1 [file bmjopen-2025-110528.reviewer_comments.pdf]

PEER REVIEW HISTORY

BMJ Open publishes all reviews undertaken for accepted manuscripts. Reviewers are asked to complete a checklist review form and are provided with free text boxes to elaborate on their assessment. These free text comments are reproduced below.

ARTICLE DETAILS

Title (Provisional)

Causes of Mortality in Humanitarian Crises Requiring Coordinated Response: A Scoping Review Protocol

Authors

Christen, Paula; Shehada, Nisreen; Abdelmagid, Nada; Ratnayake, Ruwan; Abukmail, Hanan; Hamlet, Arran; McGowan, Catherine; Watson, Oliver J; Rao, Bhargavi

---

VERSION 1 - REVIEW

**Reviewer**	**1**
**Name**	**Nepogodiev, Dmitri**
**Affiliation**	**University of Birmingham, Academic Department of Surgery**
**Date**	**03-Dec-2025**
**COI**	**None**

This systematic review protocol will address an important but understudied area. However, I feel that the study as currently presented is too broad. The authors propose to explore a very wide range of disruptions including conflict, natural disasters, drought, etc. If the focus were on how these crises impact health systems and thus mortality there might be a clear narrative thread, but it seems the authors also want to look at numbers and causes of direct deaths related to these disasters. I'm not sure that's particularly useful as clearly the impact of armed conflict will be very different to a drought. I believe a tighter remit (perhaps around health systems) would be more meaningful and impactful.

Additional comments

-Inclusion and exclusion criteria would be easier to follow with a single bullet pointed list of inclusion and another single list of exclusion criteria rather than trying to fit it to a PICO structure.

-The approach to abstract selection is problematic. The authors assert that a LLM-assisted approach will have higher sensitivity that human abstract review. I don't think there is any

broad consensus that this is currently true. The authors say they will pilot the LLM-assisted approach defining good agreement as a as a ≥98% consensus with human review but it isn't clear how many abstracts will be included in this pilot (what sample size is needed to conclusively demonstrate 98& agreement?). The authors also say LLM decisions will be cross-checked by humans but it isn't clear what this means in practice - are they effectively saying the LLM is a second reviewer with a human going through all the abstracts in the normal way? It is unclear whether LLMs will do full text review. This is a major issue - currently the abstract selection strategy would lead to low confidence in its results.

-Abstract methods say LLM will be used for data extraction but the main manuscript says this will be done by the authors

-Introduction is long. Please reduce to three key paragraphs. Please avoid over using ChatGPT. The unusually large number of subordinate clauses and emdashes is distracting.

-Ensure all terms used are defined - e.g. acute versus protracted conflict.

-What crtieria/list is used to identify LMICs.

**Reviewer**	**2**
**Name**	**Goldblatt, Peter**
**Affiliation**	**University College London, UCL Institute of Health equity**
**Date**	**09-Mar-2026**
**COI**	**None**

This protocol proposes a very wide ranging review of literature on causes of death in recent humanitarian crises. While it is appropriate, in the face of an explosion of recent crises, to try to limit the scope of the review to something that is manageable, there are inconsistencies in the definition of scope. This risks either unintended widening of the review or arbitrary, unintended curtailment of scope.

Detailed comments

Introduction, first sentence

This sentence identifies humanitarian crises becoming more violent in recent years. While some of the increase is characterised in this way, this description contradicts later text focusing on the increase associated purely with natural disasters as a result of climate change. It gives a misleading impression of the justification and scope of the proposed review as described in paras one and two of the section on "gaps in the literature"

Inclusions and exclusions - populations

These need to be more clearly/tightly defined. Crisis affected populations will frequently be displaced to become internal or international migrants or refugees (see Table 1). However,

the exclusion criteria would seem to exclude these populations if they have been displaced to non-crises areas such as Lagos or to high income countries (e.g. deaths in boat crossings between high income countries or in camps such as Calais). The inclusion criteria needs to be more clearly spelled out to align with the exclusion criteria.

Inclusions and exclusions - outcomes

The age groups selected appear very restrictive. Aside from crude rates (which can be very misleading), they seem to be restricted to mothers and children in the six years after conception of the child. What is missing are age-specific death rates e.g. adolescents, young men and women , the elderly. In rare cases, papers may also include various forms of age-standardised rates, but these seem to be excluded.

Inclusions and exclusions - other inclusions

The proposal to consider all languages needs to be more clearly defined in terms of capacity (see the need to draw on work by multilingual collaborators). The number of potential papers/reports that they can cope with must surely be limited.

Table 1 Data extraction plan - Method used to ascertain cause of death

The use of only household members to collect retrospective autopsy reports could be problematic in a crisis where all members (or at least adults) may have been killed/died.

Table 1 Data extraction plan - Categories used to ascertain cause of death

The contrast between direct conflict related deaths and non-violent deaths excludes many violent deaths experienced by refugees and migrants from a crisis (accidents in travelling, deaths from homicide or related to human trafficking).

Data synthesis and analysis

As well as drawing comparators from original databases such as DHS, MICS, the researchers should consider other collated databases which have cleaned up and harmonised raw data e.g. WHO Mortality database, Health Equity Assessment Toolkit, UNICEF child data.

VERSION 1 - AUTHOR RESPONSE

Reviewer: 1

Dr. Dmitri Nepogodiev, University of Birmingham

Comments to the Author:

This systematic review protocol will address an important but understudied area. However, I feel that the study as currently presented is too broad. The authors propose to explore a very wide range of disruptions including conflict, natural disasters, drought, etc. If the focus were on how these crises impact health systems and thus mortality there might be a clear narrative thread, but it seems the authors also want to look at numbers and causes of direct deaths related to these disasters.

I'm not sure that's particularly useful as clearly the impact of armed conflict will be very different to a drought. I believe a tighter remit (perhaps around health systems) would be more meaningful and impactful.

Response:

We thank the reviewer for this thoughtful comment and for recognising the importance of the topic.

We agree that humanitarian crises are heterogeneous and that mortality patterns differ substantially across crisis types, including armed conflict, natural disasters, and climate-related events. The intention of this review is to address a key gap in the literature: the lack of a comprehensive synthesis of cause-specific mortality across diverse humanitarian contexts.

Our review is explicitly framed within the conceptual model proposed by Checchi et al., 2017[1], in which trigger events (armed conflict, earthquakes, floods, drought, and other shocks) give rise to crisis-emergent risk factors, which in turn shape health outcomes and mortality impacts. While trigger events themselves are highly heterogeneous, they converge on a common set of mediating risk factors, including disruption of routine health services and reduced healthcare access and quality, deterioration of water, sanitation, and hygiene (WASH) conditions, and compromised food security and nutrition. These shared pathways provide the unifying narrative thread for our review. What varies across crisis types is not the fundamental structure of the pathway from trigger to mortality, but the relative weight each risk factor carries and the balance between direct and indirect causes of death. Characterising this variation is a central objective of the review and is precisely what a comparative synthesis is positioned to deliver.

We respectfully note that focusing on a single mechanism, such as health system disruption, would capture only part of this pathway and would miss the interdependencies between health services, WASH, and nutrition that jointly determine mortality in most crises. In acute conflict or sudden-onset disasters, mortality may be driven substantially by direct causes such as injuries or violence, whereas in protracted or slow-onset crises, indirect causes mediated through the risk factors above may predominate. Understanding both direct and indirect causes, and how their relative contributions and underlying risk-factor profiles vary across crisis types, is central to informing effective humanitarian response.

To address concerns regarding breadth and interpretability, we have strengthened the manuscript in three areas:

(i) Introduction:

We have revised the Introduction to explicitly frame the review within the Checchi et al. (2017) trigger event → emergent risk factor → outcome → impact model. We now articulate how diverse trigger events converge on a common set of risk factors (health service disruption, WASH, food security and nutrition), while their relative contributions, and the balance between direct and indirect causes of death, vary by crisis type. This framing

[1] http://dx.doi.org/10.1016/S0140-6736(17)30702-X

provides a clearer narrative thread and a stronger rationale for a comparative synthesis across crisis contexts.

(ii) Aims (Objective 3):

We have revised the objective to explicitly reflect this distinction:

> *"Distinguish between direct and indirect causes of mortality and characterise how their relative contributions shift along the acute-to-protracted crisis spectrum and across underlying risk-factor pathways (health services, WASH, food security and nutrition)."*

(iii) Data synthesis and analysis (Synthesis section):

We have clarified how heterogeneity across crisis types will be addressed:

> *"We will categorise studies by crisis context (e.g., armed conflict, natural disasters, and climate-related crises) to summarise the characteristics of included studies, critical appraisal scores, relative causes of death, and other key findings. Given the heterogeneity of crisis types, findings will be stratified by crisis context to ensure meaningful interpretation. Results will be presented narratively and, where appropriate, using tables and figures."*

These changes address the reviewer's concerns by strengthening the conceptual framework, clarifying the analytical approach, and ensuring that differences across crisis types are explicitly accounted for.

See tracked changes in the Introduction, Aims, and Data synthesis sections.

Additional comments

Inclusion and exclusion criteria would be easier to follow with a single bullet pointed list of inclusion and another single list of exclusion criteria rather than trying to fit it to a PICO structure.

<u>Response:</u>

Thank you for this helpful suggestion. We agree that the original presentation of the inclusion and exclusion criteria may have reduced readability.

In response, we have revised the manuscript to present the eligibility criteria as two consolidated bullet-point lists: one for inclusion criteria and one for exclusion criteria.

This revised format improves clarity and accessibility for readers while maintaining consistency with the review objectives.

The updated eligibility criteria are now presented in the Methods section under "Inclusion and exclusion criteria."

See tracked changes in the Methods section.

-The approach to abstract selection is problematic. The authors assert that a LLM-assisted approach will have higher sensitivity that human abstract review. I don't think there is any broad consensus that this is currently true. The authors say they will pilot the LLM-assisted approach defining good agreement as a as a ≥98% consensus with human review but it isn't clear how many abstracts will be included in this pilot (what sample size is needed to conclusively demonstrate 98& agreement?). The authors also say LLM decisions will be cross-checked by humans but it isn't clear what this means in practice - are they effectively saying the LLM is a second reviewer with a human going through all the abstracts in the normal way? It is unclear whether LLMs will do full text review. This is a major issue - currently the abstract selection strategy would lead to low confidence in its results.

Response:

Thank you for this comment. We agree that the original description of the LLM-assisted screening approach lacked sufficient clarity and could raise concerns regarding validity and reproducibility. We have revised the Methods section to address these issues. We also frame this as an opportunity to learn about LLM-assisted screening, as per one of the authors' previous work in this area.

First, we have removed the assertion that LLM-assisted screening has higher sensitivity than human review, as we agree that there is currently no broad consensus supporting this claim. Instead, we now frame the LLM as a screening support tool whose performance is context-dependent and requires validation.

Second, we have clarified the pilot phase and agreement threshold. Specifically, we now describe a validation step in which LLM performance is assessed against human decisions on a predefined random sample of abstracts, with the ≥98% concordance threshold used as a pragmatic benchmark to iteratively refine the screening approach prior to full implementation. We have also specified the sample size for this pilot.

Third, we have substantially clarified the role of the LLM in the screening workflow. The revised manuscript now makes explicit that the LLM is used for initial title and abstract screening only, after validation, and does not replace human judgement. Records classified as "include" and those deemed uncertain are reviewed by human reviewers. We have also clarified that conservative inclusion thresholds are applied to minimise the risk of missed eligible studies.

Fourth, we have clarified that full-text screening is conducted independently by two human reviewers, and that the LLM is not used at this stage.

Finally, we have revised the text to more transparently acknowledge the limitations of LLM-assisted screening, including the potential for misclassification and the importance of human oversight.

We believe these revisions substantially improve the clarity, transparency, and methodological rigour of the screening approach.

- Abstract methods say LLM will be used for data extraction but the main manuscript says this will be done by the authors

<u>Response:</u>

Thank you for highlighting this inconsistency. We have corrected the discrepancy between the abstract and the main manuscript. Data extraction will be conducted manually by the authors, and references to the use of LLMs for this purpose have been removed from the abstract to ensure consistency throughout the manuscript.

- Introduction is long. Please reduce to three key paragraphs. Please avoid over using ChatGPT. The unusually large number of subordinate clauses and emdashes is distracting.

<u>Response:</u>

We thank the reviewer for this helpful comment. We have revised the Introduction to improve clarity, readability, and writing style. The section has been condensed into three focused paragraphs, with simplified sentence structure, removal of em dashes, and a more direct academic tone.

(i) Reduction to three key paragraphs

The Introduction has been restructured into three concise paragraphs to improve focus and logical flow. These now cover: (i) the context and complexity of humanitarian crises, (ii) gaps in the evidence base, and (iii) the need for a comprehensive synthesis of cause-specific mortality. This restructuring reduces repetition and improves readability.

(ii) Simplification of sentence structure and tone

We have revised sentences throughout to reduce the use of subordinate clauses and improve clarity. For example:

Before:

> *"Despite the increased complexity and evolving nature of crises, the evidence base underpinning humanitarian decision-making remains limited and often fragmented, and may not comprehensively reflect varying causes of death patterns across affected populations and contexts."*

After:

> *"Despite the increasing complexity of humanitarian crises, the evidence base informing decision-making remains limited and fragmented."*

Similarly, we simplified complex descriptive passages:

Before:

> *“Studies examining specific population groups in conflict-affected settings reveal substantial but fragmented – and often outdated – evidence on mortality patterns and causes.”*

After:

> *“Available evidence on mortality patterns and causes is substantial but fragmented and often outdated.”*

These changes reduce sentence length and improve readability.

(iii) Removal of em dashes and stylistic simplification

We have removed em dashes and revised punctuation throughout the Introduction to improve flow. For example:

Before:

> *“...substantial but fragmented – and often outdated – evidence...”*

After:

> *“...substantial but fragmented and often outdated evidence...”*

We have also revised phrasing to ensure a more natural and consistent academic tone.

(iv) Restructuring of the “Gaps in the literature” section

The section on gaps in the literature has been substantially rewritten to present a clearer and more structured argument. For example:

Before:

> *“Our preliminary searches in PROSPERO and Medline(R) did not identify any systematic or scoping review—either completed or in progress—in the past two decades...”*

After:

> *“Our preliminary searches of PROSPERO and Medline(R) did not identify any systematic or scoping review in the past two decades that has examined causes of mortality across all age groups in humanitarian crises globally.”*

We also clarified key gaps more explicitly:

> *“There is also no standardised approach to evaluating causes of death within the mortality surveys and surveillance systems commonly used in humanitarian contexts.”*

We believe these changes address the reviewer’s concerns and improve the clarity, accessibility, and overall quality of the Introduction.

- Ensure all terms used are defined - e.g. acute versus protracted conflict.

Response:

We thank the reviewer for this helpful suggestion. We have revised the manuscript to ensure that all key operational terms are clearly defined and used consistently throughout.

(i) Addition of definitions to Text box 1

We have expanded Text box 1 to include definitions of key terms used in the manuscript. We added definitions for acute and protracted crises:

> *"Acute crisis: A humanitarian crisis characterised by a sudden onset or rapid escalation over a relatively short period, often associated with immediate threats to life, health, and access to essential services."*
>
> *"Protracted crisis: A humanitarian crisis in which a significant proportion of the population remains exposed to sustained or recurrent risks over an extended period, often with prolonged displacement, disrupted services, and long-term humanitarian need."*

These additions complement the existing definitions (e.g., cause-specific mortality, all-cause mortality, excess mortality, and crisis type) and improve clarity for readers.

2. Consistency in terminology throughout the manuscript

We have also revised the wording in the Methods section to ensure consistent use of these defined terms. For example:

Before:

> *"We will categorise studies across settings (i.e. acute **conflict**, protracted **conflict**, different types of natural disasters, etc.) …"*

After:

> *"We will categorise studies across settings (e.g., acute **crises**, protracted **crises**, and different types of natural disasters) …"*

This change aligns terminology with the definitions provided in Text box 1 and avoids ambiguity.

We believe these revisions improve clarity, consistency, and interpretability of key terms across the manuscript.

See tracked changes in Text box 1 and the Methods section.

- What crtieria/list is used to identify LMICs.

<u>Response:</u>

We thank the editor for this important clarification. Included countries are **crises-affected populations in locations with an active Humanitarian Response Plan during analysis**.

Definition of eligibility criteria based on humanitarian context

We clarify that country inclusion was determined based on the presence of a Humanitarian Response Plan (HRP), reflecting settings requiring coordinated humanitarian response. The following text has been added:

> *"A predefined list of eligible countries was developed based on the presence of a Humanitarian Response Plan (HRP) during the study period. HRPs are country-level strategic plans coordinated by the UN Office for the Coordination of Humanitarian Affairs (OCHA), developed jointly by humanitarian partners in response to a crisis requiring international assistance. An active HRP indicates that a country is experiencing a humanitarian situation of sufficient scale and severity to warrant a coordinated international response, and therefore serves as a practical, widely recognised marker of countries in crisis."*
>
> *"Eligible studies will include populations in countries with an active HRP at the time of data collection."*
>
> *"This approach identifies settings requiring coordinated humanitarian response."*

(i) Explicit listing of eligible countries

To improve transparency, we have provided a **supplementary table** listing all eligible countries and their corresponding years of Humanitarian Response Plan (HRP) coverage.

In addition, we have added:

"Supplementary Table 3. Countries with Humanitarian Response Plans (HRPs) and corresponding years of coverage."

These revisions ensure that both the conceptual rationale (HRP-based inclusion) is clearly described and consistently applied, thereby improving the clarity and reproducibility of the study.

See tracked changes in the Methods section and Supplementary Table 3.

Reviewer: 2
Prof. Peter Goldblatt, University College London

Comments to the Author:

This protocol proposes a very wide ranging review of literature on causes of death in recent humanitarian crises. While it is appropriate, in the face of an explosion of

recent crises, to try to limit the scope of the review to something that is manageable, there are inconsistencies in the definition of scope. This risks either unintended widening of the review or arbitrary, unintended curtailment of scope.

Detailed comments

Introduction, first sentence

This sentence identifies humanitarian crises becoming more violent in recent years. While some of the increase is characterised in this way, this description contradicts later text focusing on the increase associated purely with natural disasters as a result of climate change. It gives a misleading impression of the justification and scope of the proposed review as described in paras one and two of the section on "gaps in the literature"

Response:

We thank the reviewer for this thoughtful observation. We agree that the original wording placed disproportionate emphasis on increasing violence in humanitarian crises, which does not fully reflect the broader scope of this review.

In response, we have revised the opening paragraph of the Introduction to provide a more balanced framing of humanitarian crises, explicitly acknowledging the diversity of crisis types and drivers, including conflict, natural hazards, and climate-related events.

> *"In the past 30 years, humanitarian crises have become more complex and protracted, with diverse drivers including armed conflict, natural hazards, and climate-related events."*

This change removes the overemphasis on violence and aligns the Introduction with the full scope of the review.

See tracked changes in the Introduction (first paragraph).

Inclusions and exclusions - populations

These need to be more clearly/tightly defined. Crisis affected populations will frequently be displaced to become internal or international migrants or refugees (see Table 1). However, the exclusion criteria would seem to exclude these populations if they have been displaced to non-crises areas such as Lagos or to high income countries (e.g. deaths in boat crossings between high income countries or in camps such as Calais). The inclusion criteria needs to be more clearly spelled out to align with the exclusion criteria.

Response:

We thank the reviewer for this important comment. We agree that the original wording did not clearly specify how displaced populations were handled in relation to crisis context and geographic setting. These revisions were made to improve clarity and ensure alignment between inclusion and exclusion criteria.

We have revised the Population and setting section to explicitly define the populations included in the review. The revised text now states:

> *"Studies must examine crisis-affected populations, including internally displaced persons (IDPs), crisis-affected host populations, and other groups directly impacted by a humanitarian crisis."*

These revisions ensure that the inclusion and exclusion criteria are clearly aligned and remove ambiguity regarding the treatment of displaced populations across different settings.

See tracked changes in the Inclusion and Exclusion criteria, methods sections.
Inclusions and exclusions - outcomes

The age groups selected appear very restrictive. Aside from crude rates (which can be very misleading), they seem to be restricted to mothers and children in the six years after conception of the child. What is missing are age-specific death rates e.g. adolescents, young men and women, the elderly. In rare cases, papers may also include various forms of age-standardised rates, but these seem to be excluded.

Response:

We thank the reviewer for this important observation. We agree that the original wording of the outcome criteria may have given the impression that the review was restricted to a limited set of mortality indicators, particularly those relating to maternal and child health. These revisions were made to clarify the full scope of outcomes and ensure that all relevant age groups and mortality measures are explicitly included.

(i) Clarification of inclusion across all age groups

We have revised the outcome criteria to explicitly state that the review includes mortality outcomes across all age groups. The revised text now states:

> *"Cause-specific mortality estimates or rankings, crude and across all age groups"*

and

> *"Age-specific mortality rates, including for children under 5 years (0–4 years), children and adolescents (5–19 years), adults (20–64 years), and older adults (≥65 years)."*

This ensures that important groups such as adolescents, young adults, and older populations are clearly included.

(ii) Inclusion of a broader range of mortality measures

We have clarified that the review does not restrict inclusion based on the type of mortality metric used. The revised criteria now explicitly include:

> *"Age-standardised mortality rates, where reported"*

as well as:

> *"All-cause mortality measures, where disaggregated by cause of death"*

In addition, commonly used indicators are listed for completeness:

> *"Crude Death Rate (CDR); Under-five Death Rate (U5DR); Under-five Mortality Rate (U5MR); Infant Mortality Rate (IMR); Maternal Mortality Ratio (MMR)"*

3. Removal of implicit restrictions on methods

We have also clarified that no restrictions are placed on the methods used to estimate mortality or assign cause of death. The revised text states:

> *"No restrictions will be placed on the methods used to estimate mortality or assign cause of death."*
>
> *"Both quantitative and qualitative assessments (e.g., verbal autopsy, surveillance data, or expert attribution) will be included, provided that deaths are attributed to specific causes within a humanitarian crisis context."*

These changes ensure that a wide range of data sources and methodological approaches are captured. Overall, these revisions clarify that the review includes all relevant mortality outcomes across the life course and avoid unintended restrictions suggested by the previous wording.

See tracked changes in the Inclusion criteria (Outcomes) section

Inclusions and exclusions - other inclusions

The proposal to consider all languages needs to be more clearly defined in terms of capacity (see the need to draw on work by multilingual collaborators). The number of potential papers/reports that they can cope with must surely be limited.

<u>Response:</u>

We thank the reviewer for this important comment. We agree that the original manuscript did not sufficiently specify how non-English language studies would be managed in practice. These revisions were made to improve transparency and clarify the practical considerations and limitations related to language inclusion.

(i) Clarification of language inclusion approach

We have revised the Inclusion criteria to explicitly state our approach to language. The revised text now states:

> *"Studies published in any language."*

This reflects our intention not to restrict studies at the search stage based on language.

(ii) Description of capacity and practical implementation

We have added detail on how non-English studies will be handled during screening and data extraction:

> *"Screening and data extraction of non-English studies will be supported by multilingual members of the review team and collaborators where available."*
>
> *"Where necessary, machine translation tools (e.g., DeepL) will be used to assist with eligibility assessment and data extraction."*

(iii) Acknowledgement of capacity constraints

To address feasibility concerns, we have explicitly acknowledged that inclusion of non-English studies is dependent on available resources:

> *"Inclusion of non-English studies will be subject to available translation capacity, and any limitations in language coverage will be documented and reported."*

This ensures transparency regarding potential limitations in coverage. These revisions clarify both the intent and the practical implementation of language inclusion, while acknowledging resource constraints and ensuring that any limitations are clearly reported.

See tracked changes in the Inclusion criteria (Language) section

Table 1 Data extraction plan - Method used to ascertain cause of death

The use of only household members to collect retrospective autopsy reports could be problematic in a crisis where all members (or at least adults) may have been killed/died.

Response:

We thank the reviewer for this important observation. We agree that reliance on household members for retrospective reporting of deaths may be problematic in humanitarian crisis settings, particularly where entire households or adult members may have been displaced or killed. These revisions were made to improve the accuracy and applicability of the data extraction framework to diverse crisis contexts.

(i) Expansion of data sources for cause of death ascertainment

We have revised Table 1 to reflect a broader range of approaches used to ascertain cause of death in humanitarian settings. Specifically, we replaced the original category:

> *"Self-report by household member or other informant"*
> *"Community informant or key informant report"*
> *"Mortality surveillance system or community-based reporting system"*

These revisions ensure that the data extraction framework captures the diversity of methods used to ascertain cause of death and avoids overly restrictive assumptions about data sources.

See tracked changes in Table 1 (Data extraction plan - Method used to ascertain cause of death).

Table 1 Data extraction plan - Categories used to ascertain cause of death

The contrast between direct conflict related deaths and non-violent deaths excludes many violent deaths experienced by refugees and migrants from a crisis (accidents in travelling, deaths from homicide or related to human trafficking).

Response:

Thank you for this important observation. We agree that the original categorisation may have been overly restrictive by focusing primarily on direct conflict-related violence, and may not have adequately captured the broader range of violent deaths experienced by crisis-affected populations, particularly during displacement and migration. These revisions were made to improve conceptual clarity and ensure a more comprehensive classification of causes of death.

1. Revision of cause of death categories

We have updated Table 1 to better distinguish between different types of violent and non-violent deaths. Specifically, we replaced the original categorisation:

Before:

> *"Violent (direct conflict-related injury, blast, gunshot, etc.)"*
> *"Non-violent (indirect or disease-related)"*

After:

> *"Direct conflict-related deaths (e.g., injuries due to armed violence, blast, gunshot)"*
> *"Other external or violent causes of death (e.g., accidents, homicide, interpersonal violence, trafficking-related deaths, deaths during displacement or migration)"*
> *"Non-violent causes of death (e.g., communicable diseases, non-communicable diseases, maternal conditions, malnutrition)"*

This revised structure separates direct conflict-related mortality from other forms of violence and external causes, ensuring that deaths occurring during displacement or migration (e.g., accidents, trafficking, or interpersonal violence) are appropriately captured.

See tracked changes in Table 1 (Data extraction plan - Categories used to ascertain cause of death).

Data synthesis and analysis

As well as drawing comparators from original databases such as DHS, MICS, the researchers should consider other collated databases which have cleaned up and harmonised raw data e.g. WHO Mortality database, Health Equity Assessment Toolkit, UNICEF child data.

Response:

We thank the reviewer for this helpful suggestion. We agree that additional harmonised and collated data sources can provide valuable context for interpreting mortality patterns and strengthen the robustness of comparisons. These revisions were made to expand the range of comparator datasets and improve the comprehensiveness of the analysis.

Expansion of comparator data sources

We have revised the Data synthesis and analysis section to include additional harmonised and collated databases, alongside the originally listed sources. Specifically, we replaced the original text:

Before:

> *"Key reference sources will include the Demographic and Health Surveys (DHS), Multiple Indicator Cluster Surveys (MICS), World Bank population and health statistics, the Global Burden of Disease (GBD) study, and other comparable national datasets."*

After:

> *"Key reference sources will include the Demographic and Health Surveys (DHS), Multiple Indicator Cluster Surveys (MICS), World Bank population and health statistics, and the Global Burden of Disease (GBD) study. Where appropriate, we will also draw on harmonised and collated datasets such as the WHO Mortality Database, the Health Equity Assessment Toolkit (HEAT), and UNICEF child health and mortality datasets, as well as other comparable national and international data sources."*

These additions ensure that the analysis can incorporate standardised and comparable data sources that have undergone harmonisation and quality checks, thereby improving the interpretability and contextualisation of mortality estimates across settings.

See tracked changes in the Data synthesis and analysis section.

Reviewer: 1
If you have selected 'Yes' above, please provide details of any competing interests.: None

Reviewer: 2
If you have selected 'Yes' above, please provide details of any competing interests.: Not applicable'

VERSION 2 - REVIEW

**Reviewer**	**2**
**Name**	**Goldblatt, Peter**
**Affiliation**	**University College London, UCL Institute of Health equity**
**Date**	**23-Jun-2026**
**COI**	

The authors have addressed the reviewers comments
