# Supplementary material for: Causes of mortality in humanitarian crises requiring coordinated response: a scoping review protocol
Source: BMJ Open. 2026 Oct 7;16(10):e110528. doi: 10.1136/bmjopen-2025-110528 (PMC13646983; doi:10.1136/bmjopen-2025-110528)
Supplement: Supplementary file 2 [file bmjopen-16-10-s001.docx]

# **Causes of Mortality in Humanitarian Crises Requiring Coordinated Response: A Scoping Review Protocol**

***Supplement***

**Supplemental Table 1.**  PROSPERO search terms

disasters OR humanitarian crisis OR complex emergency AND mortality or mortality rate AND Epidemiologic OR Systematic Review OR Meta-Analysis OR Review of reviews

*Note*: In total, the search yielded **135** potentially relevant PROSPERO-registered systematic reviews focused on health in the context of humanitarian crises and disasters. Of these:

- 10 reviews focused on natural disasters (e.g. earthquakes, floods, storms) and their health impacts.
- 7 reviews examined health system disruptions, including hospital evacuation, emergency department overcrowding, and referral mechanisms.
- 9 reviews addressed maternal, neonatal, or reproductive health in crisis settings.
- 6 reviews focused on nutritional interventions or acute malnutrition in children.
- 11 reviews explored mental health and psychosocial outcomes, including PTSD, bereavement, and suicide.
- 5 reviews examined the impacts of climate change or environmental events on health outcomes.
- 4 reviews evaluated the health of refugees, migrants, or displaced populations.
- 6 reviews investigated the efficacy of public health or clinical interventions in humanitarian settings (e.g. drills, triage models, e-health, MDA campaigns).
- 8 reviews focused on infectious diseases, vector-borne diseases, or disease outbreaks following disasters.
- 5 reviews analyzed surgical or trauma care in emergency or conflict-affected settings.

**Supplemental Table 2**

| **Theme** |  | **MEDLINE** | **Search results** |
| --- | --- | --- | --- |
| **Disasters (All hazards)** | 1 | exp Disasters/ or disaster*.mp  Displacement  Refugee  cris*s | 138632 |
| **Armed Conflict and Political Violence** | 2 | (conflict* not conflicting).mp. or Armed Conflicts/ or war.mp. or exp War Crimes/ or genocide.mp. | 175702 |
| **Food Insecurity and Malnutrition** | 3 | malnutrition.mp. or malnutrition/ or severe acute malnutrition/ or starvation/ or Famine/ or famine.mp. | 81614 |
| **Humanitarian Response** | 4 | exp Relief Work/ or relief work*.mp. | 6835 |
| **Humanitarian Crisis** | 5 | (humanitarian cris*s or humanitarian*).mp. | 8855 |
| **Epidemics and Disease Outbreaks** | 6 | Epidemics/ or epidemic*.mp. or Disease Outbreaks/ | 232154 |
| **Outcome** | 7 | exp Death/ or (death or mortality* or fatalit* or casualt*).mp. or exp Mortality/ | 2581973 |
|  | 8 | 1 or 2 or 3 or 4 or 5 or 6 | 620092 |
|  | 9 | 7 and 8 | 79383 |
| **Time** | 10 | limit 9 to yr="2015 -Current" | **38137** |

**Supplementary Table 3. Countries with Humanitarian Response Plans (HRPs) and corresponding years of coverage**

| **Country** | **HRP Coverage (Years)** |
| --- | --- |
| Afghanistan | 2016–2025 |
| Burkina Faso | 2020–2025 |
| Burundi | 2021 |
| Cameroon | 2018–2025 |
| Central African Republic | 2015–2025 |
| Chad | 2015–2025 |
| Colombia | 2020–2025 |
| Democratic Republic of the Congo | 2015–2025 |
| El Salvador | 2021–2025 |
| Ethiopia | 2016–2025 |
| Guatemala | 2021–2025 |
| Haiti | 2016–2025 |
| Honduras | 2021–2025 |
| Iraq | 2015–2021 |
| Kenya | 2015 |
| Libya | 2015–2021 |
| Madagascar | 2015 |
| Mali | 2016–2025 |
| Mauritania | 2016 |
| Mozambique | 2015–2025 |
| Myanmar | 2016–2025 |
| Niger | 2016–2025 |
| Nigeria | 2016–2025 |
| Occupied Palestinian Territory | 2017–2023 |
| Pakistan | 2021 |
| Peru | 2016 |
| Republic of the Congo | 2017 |
| Senegal | 2017 |
| Somalia | 2018–2025 |
| South Sudan | 2018–2025 |
| Sudan | 2018–2025 |
| Syrian Arab Republic | 2018–2025 |
| Ukraine | 2018–2025 |
| Venezuela (Bolivarian Republic of) | 2019–2024 |
| Yemen | 2018–2025 |
| Zimbabwe | 2019–2021 |

HRP = Humanitarian Response Plan.

Years indicate periods during which a country had an active Humanitarian Response Plan.

**Supplementary Table 4.** Data extraction plan

| **Source** | - Report - ID - Study ID - Author ID - Citation details |
| --- | --- |
| **Eligibility** | - Eligibility and exclusion criteria |
| **Methods** | **Study design and/or data collection method**   - Cross-sectional mortality survey - Other observational study (e.g., cohort) (specify) - Community-based mortality surveillance - Health management information system - Capture-recapture - Repeat census - Census - Mathematical modelling - Other routine data collection system (specify) - Other (specify)   **Data collection time period**   - Start and end dates (if available) - Duration of data collection   **Method used to ascertain cause of death**   - Self-report by household member or other informant - Community informant or key informant report - Mortality surveillance system or community-based reporting system - Verbal autopsy - Review of hospital records - Review of death certificates - Other (specify)   **Categories used to ascertain cause of death**   - **Direct conflict-related deaths:** (e.g., injuries due to armed violence, blast, gunshot) - **Other external or violent causes of death:** (e.g., accidents, homicide, interpersonal violence, trafficking-related deaths, deaths during displacement or migration) - **Non-violent causes of death:** (e.g., communicable diseases, non-communicable diseases, maternal conditions, malnutrition)   **Evaluation of mortality estimation attributes**   - Whether a formal evaluation was conducted - Attributes assessed (e.g., timeliness, sensitivity, positive predictive value, simplicity, acceptability) - Summary of findings from the evaluation - Not mentioned   **Potential reporting bias of mortality estimates**   - Any mention of potential biases in reporting mortality data (free text) - Type of bias described (e.g., under-reporting, over-reporting, misclassification) - Context in which bias occurs (e.g., conflict setting, cultural factors, political pressures, security/access constraints) - Hypothesised reasons for bias (e.g., recall period, survey design, reluctance to report certain deaths) - Whether authors compared reported figures to alternative data sources to assess bias |
| **Setting** | **Population representativeness**   - Yes/No   - If yes, which population does it represent   **Intended scale and geographical coverage**   - National, sub-national, or local - Specific geographic area(s) covered   **Population type**   - Internally displaced persons - Refugees - Crisis-affected host population - Nomadic or pastoralist communities - Mixed (specify)   **Setting type**   - Camp (e.g., refugee or internally displaced persons) - Rural or remote - Urban - Mixed rural/urban   **Primary crisis driver**   - Conflict or armed violence - Natural disaster (e.g., earthquake, tsunami) - Climate-related crisis (e.g., drought, flood, cyclone, heatwave) - Other (specify)   **Secondary crisis driver(s)**   - Conflict or armed violence - Natural disaster (e.g., earthquake, tsunami) - Climate-related crisis (e.g., drought, flood, cyclone, heatwave) - Other (specify)   **Crisis duration**   - Acute crisis - Protracted crisis   **Presence of environmental stressors**   - Yes: explicitly mentioned (e.g., drought, flooding, cyclone) - No: not mentioned |
| **Population and exposure** | **Total number of individuals**   - Overall sample size - Breakdown by relevant subgroups (if available)   **Sociodemographic characteristics**:   - **Age** (e.g., age ranges, mean/median, or specific focus such as children under five or older adults) - **Sex** (male, female, other if reported) - **Types of population groups included** (e.g., internally displaced persons, refugees, host communities, general population) - **Country and sub-national area** (as defined in the study) - **Socioeconomic status** (measured or described by income, education, occupation, asset index, etc.) - **Other stratifications** (disability status, prior comorbidities, ethnic or linguistic minority status, or other characteristics that may be associated with disproportionate mortality in crises) |
| **Comparison group** | - Same population but different time period (e.g., mortality before displacement or onset of crisis) - Different population in a comparative observational study (e.g., two cohorts, one crisis-affected, one non-affected) - *If present:* Record description of comparator population and how it differs from crisis-affected group.   **Method used to determine comparison status**   - Description of how the comparator group was selected or defined (e.g., matched on region, age, sex, or socioeconomic indicators). |
| **Outcomes** | **Primary outcomes to be extracted**:   - **All-cause mortality**   - Total number of deaths and/or mortality rate within the study population   - If available, extract standard indicators, such as: - Crude Death Rate (CDR) - Under-five Death Rate (U5DR) - Under-five Mortality Rate (U5MR) - Infant Mortality Rate (IMR) - Maternal Mortality Ratio (MMR) - **Cause-specific mortality**   - Mortality attributed to specific causes   - Further disaggregated as follows:     - **Natural causes** (e.g., communicable or non-communicable diseases; ICD-coded)     - **Non-natural causes** (e.g., injuries, violence, accidents; ICD-coded)     - **Specific categories** of interest (e.g., maternal mortality, under-five mortality, malnutrition-related deaths) - **Years Lived with Disability (YLD)**   - If available, estimates of non-fatal disease burden - **Population Attributable Risk (PAR)**   - Estimates of the proportion of deaths attributable to specific exposures or conditions, if reported. |
| **Estimate of effect/results** | **Effect measures and qualitative accounts of mortality:**   - Quantitative effect measures (if reported):   - Relative risk / Risk ratio of relative causes of death   - Hazard ratio (HR) of relative causes of death   - Odds ratio (OR) of relative causes of death   - 95% confidence intervals (CIs) for all effect estimates   - Standardised Mortality Ratio (SMR)   - Person-time data (e.g., person-years at risk) - Descriptive estimation of mortality:   - Rankings of leading causes of death     - Based on reported primary, secondary, or tertiary causes |
| ICD: International Classification of Diseases | |
